# Cross-border healthcare-seeking and utilization behaviours among ethnic minorities: exploring the nexus of the perceived better option and public health concerns

**DOI:** 10.1186/s12889-024-18981-1

**Published:** 2024-06-04

**Authors:** Sik Yee Leung, Hok Bun Ku

**Affiliations:** Department of Applied Social Sciences, The Hong Kong Polytechnic University, Hong Kong, China

**Keywords:** Medical tourism, Global equity in health care, Inequalities in accessing to healthcare among ethnic minorities in Hong Kong

## Abstract

**Background:**

Many ethnic minorities in Hong Kong seek medical tourism after encountering inequalities in access to local healthcare because of language barriers and cultural-religious differences. The present study explored the ethnic minorities’ lived experiences of medical tourism and issues arising from cross-border health-seeking relevant to this specific population.

**Methods:**

Qualitative in-depth interviews with 25 ethnic minority informants from five South Asian countries in 2019.

**Results:**

The 19 informants out of the 25 have sought assistance from their international networks for home remedies, medical advice and treatments of traditional/Western medicines, for they are more costly or unavailable in Hong Kong and for issues related to racial discrimination, language barriers, transnationalism engagement, cultural insensitivity, and dissatisfaction with healthcare services in Hong Kong.

**Discussion:**

Medical tourism can relieve the host country’s caring responsibilities from healthcare services, so the government might no longer be hard-pressed to fix the failing healthcare system. Consequently, it could cause public health concerns, such as having patients bear the risks of exposure to new pathogens, the extra cost from postoperative complications, gaps in medical documentation and continuum of care, etc. It also triggers global inequities in health care, exacerbating unequal distribution of resources among the affordable and non-affordable groups.

**Conclusion:**

Ethnic minorities in Hong Kong sought cross-border healthcare because of structural and cultural-religious issues. The surge of medical tourism from rich and developed countries to poor and developing countries may infringe upon the rights of residents in destination countries. To mitigate such negative impacts, policymakers of host countries should improve hospital infrastructure, as well as train and recruit more culturally sensitive healthcare workers to promote universal health coverage. Healthcare professionals should also strive to enhance their cultural competence to foster effective intercultural communication for ethnic minority groups.

**Supplementary Information:**

The online version contains supplementary material available at 10.1186/s12889-024-18981-1.

## Background

Although the global medical tourism market experienced a decline of 46.9% in 2020 during the COVID-19 pandemic, it still reached a market size of USD11.56 billion [1]. It is predicted to rebound and grow significantly, reaching USD53.51 billion by 2028. Its fast growth may have implications on destinations’ healthcare systems and public health outcomes. For example, medical tourism can create a highly specialized private healthcare system in destination countries for medical tourists and wealthier domestic patients [2]. However, it is also argued [3] that patients who experienced medical disenfranchisement for appropriate treatment in their host countries because of racial and cultural discrimination may be driven to seek cross-border medical and health care. Thus, their experience of medical disenfranchisement in the host country exacerbates health inequalities among local residents, especially their counterparts and the less affluent, in the destination countries.

Medical-health-wellness tourism refers to ‘a branch of tourism in general in which people aim to receive specific treatments or seek an enhancement to their mental, physical, or spiritual well-being’ [4]. It primarily involves medical treatments or invasive procedures such as surgeries, whereas health and wellness tourism offer activities, such as Ayurveda therapies, spa treatments, pilates retreats, procuring herbal or homoeopathic medicine, etc. to boost physical, mental and spiritual fitness. Medical tourism focuses on maintaining, enhancing, and restoring an individual’s well-being in both body and mind [5]. While it was considered a new global niche market approximately 20 years ago [6], it has since evolved into a global phenomenon. It continues to gain popularity, for it addresses patients’ unmet health needs by providing high-quality or timely services that may not be available or less costly than their host countries.

Literature has suggested that seeking cross-border health care can disrupt the continuity of patient care [7]. To minimise the harm, public health practitioners could play a dual role in medical tourism: assisting patients in identifying suitable healthcare facilities overseas and alerting them to potential risks and ethical/legal issues in seeking treatments abroad [8]. Healthcare providers must warn patients of potential health hazards and challenges when helping them make informed decisions about their care. Studies [7, 8] have suggested that patients seeking medical tourism are for more favourable costs, wait times, quality of services, care and healthcare facilities, language accessibility, religious considerations, political climate, convenience and affordability. However, engaging in cross-border treatments in foreign countries could submit individuals and the public to certain health risks, namely contracting infections and exposing to biosecurity risks abroad, such as nosocomial infections, post-surgical complications, and exposure to new pathogens, microbiological fauna and flora [9, 10]. Medical tourism will also contribute to disparities in health care by exacerbating the unequal distribution of resources and accelerating the brain drain from public hospitals to private counterparts in destination countries [11]. Research on medical-health-wellness tourism among diaspora travellers has already raised concerns about global inequity when they engage in multiple healthcare systems in their host countries and the destination countries. This current study reveals that ethnic minorities may exploit healthcare services in poorer and developing countries for lower costs.

In order to explore the relationship between medical tourism and equity and its implications for public health, a literature search in the databases PubMed and JSTOR from January 2014 to December 2023 was conducted, which found 31 articles in PubMed and 5 articles in JSTOR. Past research focused on ethical implications, service quality, sensitive practice, destinations, and medical tourism marketing, as well as issues of globalization and policies on international patients. Studies have argued that the growth in medical tourism may exacerbate inequities in the global distribution of health and wealth [12]. Policies that raise people’s satisfaction and trust towards the healthcare system and treatment may reduce people’s need for medical tourism [13]. In brief, there are issues related to medical tourism, such as its unclear impact on the healthcare system and ethical concerns on the implications for public health in destination countries; hence, national and worldwide studies are called for on medical tourism [14]. With that in mind, the problem statement of the current study addresses the rapid growth of international medical tourism that significantly impacts global public health, contributing to health disparities and unequal distribution of resources. Therefore, the present study aims to explore the lived experiences of cross-border healthcare seeking/utilization behaviours among ethnic minorities in Hong Kong, as well as issues arising from medical tourism relevant to this specific population.

## Aim and design

The qualitative study adopted three data collection methods, (a) face-to-face individual in-depth interviews to explore ethnic minorities’ lived experiences in seeking and utilizing health care outside Hong Kong and their home countries, (b) unstructured observations in informants’ homes observing and listening quietly while they were seeking and utilizing healthcare services, and (c) unstructured informal dialogues with ethnic minorities groups in the religious/service centres. The study was conducted from June 2019 to September 2019 in Hong Kong.

### Sample and setting

In this study, ethnic minority informants from five South Asian countries, who represented the largest ethnic minority population in Hong Kong, were selected using snowball sampling with six inclusion criteria: (1) Being an Indian, Pakistani, Bangladeshi, Sri-Lankan or Nepali by ancestry; (2) 18 + years old male or female Hong Kong residents; (3) unskilled/skilled occupations; and (4) with/without Chinese or English language ability in each South Asian group. Most ethnic minorities in Hong Kong are from South Asian countries, and in order to ensure the rigour of samples, participants were mainly recruited from these five countries of South Asian countries. Prior studies [15] have shown age and gender differences in health-related behaviours ranging from preventive behaviour to treatment-seeking, self-treatment, and risky behaviour. Occupation is linked to people’s financial state, which can influence their travel participation.

As the researchers had not established any relationships with participants prior to the study’s commencement, participants were approached through the leaders of the religious/service centres. A total of 25 informants, legal residents of Hong Kong, were contacted, and none refused to participate. Their ages ranged from 21 to 76, and they lived in Hong Kong for 2 to 53 years. Sixteen out of the 25 were female. Eight were from Pakistan, 7 from Nepal, 5 from India, 4 from Bangladesh, and 1 from Sri Lanka. All interview sessions concluded once the data was saturated, i.e. at the point when no new themes emerged from additional data after interviewing all informants. The informants’ profiles, including their sex, age, ethnicity, marital status, religion, family role, household composition, education level, language skills, occupational class, monthly household income, and length of stay in Hong Kong, were listed in Table 1 (All names are pseudonyms).

**Table 1:** Informants’ profile

Informant	Pseudonyms	Ethnicity	Religion	Gender	Age	Years of stay in Hong Kong	Language
1	Bushra	Pakistani	Muslim	F	41	14	Urdu, Hindi, Punjabi, English, Chinese
2	Farhan	Pakistani	Muslim	F	30	20	Urdu, Punjabi, English, Chinese
3	Anik	Bangladeshi	Muslim	M	51	25	Hindi, Begali
4	Sneha	Indian	Sikh	F	34	11	Punjabi, Hindi
5	Gulbadan	Pakistani	Muslim	F	28	2	Urdu, English
6	Manish	Pakistani	Muslim	F	68	14	Urdu, English
7	Sharif	Pakistani	Muslim	M	74	53	Urdu, Punjabi
8	Amrita	Pakistani	Muslim	F	40	16	Urdu, Punjabi
9	Umar	Pakistani	Muslim	F	36	36	Punjabi, English
10	Alisha	Nepali	Buddhist	F	40	22	Nepali
11	Chaha	Nepali	Buddhist	F	42	10	Nepali
12	Rafiq	Pakistani	Muslim	M	72	13	Urdu, English
13	Alina	Nepali	Buddhist	F	39	10	Nepali
14	Heena	Nepali	Buddhist	F	33	7	Nepali
15	Chantin	Nepali	Christian	F	34	2	Nepali, English
16	Mayra	Indian	NA	F	21	21	Urdu, English, Chinese
17	Bilhana	Nepali	Hindu	F	30	6	Nepali, English
18	Sadiya	Bangladeshi	Muslim	F	44	9	Begali, Hindi, Urdu, English
19	Yash	Indian	Hindu	M	42	9	Tamil, English
20	Sang	Nepali	Hindu	M	34	2.5	Nepali, English
21	Raj	Indian	Muslim	M	74	53	Tamil, English
22	Minhaj	Bangladeshi	Muslim	M	54	30	Begali, Hindi, Urdu, English
23	Barsha	Bangladeshi	Muslim	F	49	24	Begali, Hindi, English
24	Lakma	Sri Lankan	Islam	M	75	49	Sri Lankan, Tamil, English
25	Taksh	Indian	Muslim	M	76	53	Tamil, Hindi, English

### Data collection and data analysis

The interview guide for the present study, pilot-tested before use, comprises questions about ethnic minorities’ healthcare needs, the factors influencing their health-seeking and healthcare utilization, and their reasons and patterns for seeking cross-border health care. The first author, a female academic in nursing with a doctoral degree, conducted the interviews in English at the religious/service centres, and interpreters with proper interpretation training were arranged if respondents did not speak English. Each interview was audio-recorded and about 90 min.

The current study adopted the Four C’s model of thematic analysis [16], identifying codes (C) from respondents’ statements, clustering and grouping of codes into categorises (C), mapping the categories identify their connectivity (C) and relationality, and constructing (C) the global theme using the retroductive reasoning. The first author carefully read through the interview transcripts, sentence by sentence, to get an overview of the data. NVivo 12 Pro was used to store the inductive codes of specific themes to generate relevant parent and child nodes for the research aim. From the side-by-side comparison of those nodes, key themes were developed, capturing the meanings of participants’ lived experiences.

The trustworthiness of the study’s quality was ensured through reflexivity, peer debriefing, and peer examination. In order to eliminate researchers’ assumptions, preoccupations, and stereotypical views on the research topic, the authors critically reflected on their prior knowledge, perceptions, and clinical experiences. Their discussion was insightful and rewarding for data interpretation. To test the trustfulness and verify her understanding and interpretations of the findings, the first author compared the results from different data sources and cross-checked the findings with her fieldnotes. Finally, the second author was invited to discuss with the first author the categories, codes, themes, and interpretations; together, they reviewed and verified the results.

### Ethical considerations

The Ethics Committee of the relevant University granted the present study ethical approval. As a non-clinical project, all the data were collected via interviews with mentally intact adult participants who gave written informed consent. They were briefed about the study’s aims and methods and participated voluntarily, knowing they had the right to withdraw at any time, without penalty or consequences.

## Results

The 19 informants out of the 25 admitted having engaged in medical-health-wellness tourism outside Hong Kong or their home countries. Their ages ranged from 21 to 76, and they lived in Hong Kong for two to 53 years (Table 1). The results using the Four C’s model of thematic analysis (Appendix 3) were presented as follows:

### Theme 1. Pre-service: decision-making and choices in healthcare utilization

#### Seeking health information from respondents’ connections in their home country

Transnational health-seeking is common among South Asian groups in Hong Kong. The cultural and social context and family ties among immigrants can influence their decisions and choices of healthcare utilization [17]. Sadiya said if she is still not feeling well after taking medicine prescribed by her Hong Kong doctor, she wants to look for a better option; she would ask her mother to seek advice from her uncle in Bangladesh, check the efficacy of her medicine or inquire for a better option:

Health information definitively we can get from out… I mean, yeah, but maybe we have some family back in Bangladesh. Yeah, we have. So, if I I’m feeling bad or maybe not satisfied with the medicine or I want to recheck that is the good one. It is the appropriate one. I can check all that. I can ask my mom. Could you please ask uncle due to this condition doctor prescribed this medicine is it okay or is there anything better than this or it is the best one here that, that we can check.

The sister of Chantin is a nurse in India so she will ask her how to treat their health problems if she or her child gets sick:

My sister is in India, my younger sister and she is also a nurse. And, uh, and then sometimes if we feel discomfort, then our, if my child gets sick that I asked how to, what to do like this?’

The family of Minhaj is in Bangladesh, and his brother and some of his friends are practising doctors whom he turns to for any medical ailments:

I have in my family, I have my brother, he’s a doctor and also, I have some my friend in Bangladesh, they are the doctor in the different, different field. So of course I pass, I consult them because I feel that if I go to the hospital A&E, it takes us sometimes the two hours, three hours four hours.

Chaha said if her family members in Hong Kong are getting ill, they will call their family or friends back in Nepal, as they are more knowledgeable. Likewise, Heena mentioned that she is in touch with friends in Nepal through phone and Facebook.

#### Seeking health treatments which are not available in the host country

Mayra (aged 21) returns to India for medical care for its efficiency as she can just go into any clinic to get everything done and get medicine very quickly. Bushra said she brought her children back to Pakistan because the doctors who saw them last time were very good and respected them:

I brought my children back to hometown, and they a good medical person they do respect a lot.

Another in the same company of medical tourism is Minhaj, a 54-year-old Bangladeshi man. He admitted sometimes going to places like Bangkok, Thailand, for some medical services which are not available in Hong Kong:

Sometimes go to the other places also. Like Bangkok, Thailand, also I went to the, that places I… Singapore. Yea. I went there because of there is uh, a short time. I can, I had to if I get the same service in Hong Kong, but I not, I not supposed to go.

### Theme 2. Accessibility and barriers: language, waiting time, service availability, and cultural differences

#### Language barrier in seeking healthcare services

Some informants expressed having difficulty communicating with local medical professionals in Hong Kong, which drives them to seek international health care. Chantin explained that most of her friends speak very little Chinese and find it difficult to express themselves to doctors in Hong Kong, so they prefer to go back to their own country to see a doctor:

Most of the friends I heard from them, they tried to, they cannot express well that’s why, that’s why the things will be different. And then most of the friends because of they cannot express well in Hong Kong with the doctor, then they go to their own country to see the doctor. Yea, I heard many.

Sadiya remarked that it was very easy to communicate in her country of origin, contrasting her experience at a dispensary in Hong Kong. Some folks working there do not understand the language of ethnic minorities and might need to explain their problems to them several times:

Yeah. It’s a very easy to communicate and get from there. But here maybe you are going to the uh, I mean dispensary, some people don’t understand. Some people will say that what, what, what you need to explain several times this disease and this is so it’s basic like some common what we know that due to cold you need to this have this medicine, fever, this medicine and just common stuff that we collect from Bangladesh.

Farhan, a 30-year-old Pakistani housewife, explained that her country folks could use their language in their home countries so they can describe their ailments to the doctor, and some of them even opt to get their minor operations done in their home country because of the language barriers with Hong Kong healthcare providers. She explained:

But back in our country, you use our own language, so it’s better you describe everything right to the doctor, and they give you, prescribe you with the medicine. And they think that is better for them to take care, to take that medicine in country you, even though they are here in Hong Kong. And some of them they will… like if they have to do minor operation or something they prefer going back to their country because of the language barrier again.

Amrita recalled one of her visits to the hospital with a friend: when she spoke to the nurses in her native language, *‘the nurses start to laugh at them’*. Bushra criticized healthcare professionals’ level of English and said *‘if they know English, it’s easy for me to communicate with them’*.

### Cultural issue in healthcare utilization

To observe the Islamic hierarchy of health-seeking, Muslim women prefer seeking treatment from a doctor of the same faith and female, then non-Muslim and male. Amrita, a Muslim, knew she could not request a doctor of the same gender, not to mention a female doctor of the same faith. Her interpreter relayed how she dealt with the situation: *‘she just keeps on praying to god that today I should get a female doctor’.* When her daughter had some gynaecological problem, Amrita got her herbal medicine from Pakistan. Umar, also a Muslim, criticized her nurse for being culturally insensitive, as her nurse picked out a South Asian man at random in the hospital hall to be her interpreter: *‘The nurse doesn’t have that sensibility, that why she is calling a South Asian man to come and interpret’.* She, too, frequented her home country for treatment.

The above Muslim informants’ lived experiences showed that health providers’ lack of cultural competence and diversity awareness can influence ethnic minorities’ health-seeking behaviours and the utilization of cross-border health care.

### Theme 3. Post-service: experiences of health-seeking in the host society

#### Dissatisfaction with healthcare services in the host country

Barsha said that ethnic minorities are dissatisfied with the hospital service because of the long wait and shortage of doctors. Therefore, she will have it done in Bangkok, Singapore or her home country, India, for treatment when the family has time off during holidays. She revealed:

All I am saying, they are not satisfied with hospital because long time waiting, not sufficient doctors for all check-ups. So that we can have holiday time we can go Bangkok, Singapore, our countries do the check-up of our health.

Of the reasons that ethnic minorities feel dissatisfied with Hong Kong’s healthcare services, some are unique because of language, cultural and social barriers, but others are general to all residents in Hong Kong. Taksh professed that *‘a lot of red-tapism’* makes life *‘difficult’* for ethnic minorities in Hong Kong. For instance, the Hospital Authority’s inconsistent administrative arrangement for medical interpretation services is a hit-and-miss in its assignation. Both Farhan and Umar attested that they have been frequently refused by a nurse or receptionist to have a medical interpreter, while on some occasions they might get their way. For those applying for government financial assistance, they must complete multiple documents correctly; Alisha, with a language barrier, was among those *‘unable to fulfil all the addition documents’*. The government red tape is a put-off to the needy.

## Discussion

### Cross-border healthcare seeking/utilization: a personal issue

My informants, young, old, and the not-so-old, whom I met all had personal tales of discrimination about healthcare services because of their colour and race, regardless of the Hong Kong government’s pledge to healthcare equality for residents of all races and ethnicities. Numerous studies [18] agree that seeking cross-border health care can result in discrimination, social exclusion, and adverse healthcare experiences in the host country. As doctors are ethically obligated to treat patients without discrimination and with respect, Bushra’s comment is food for thought, for she has a professional nursing degree from Pakistan. Moreover, ethnic minorities may feel excluded from public services in the host country, although they are fully entitled to them. Kemppainen et al. [18] reverberate such a claim, reporting a higher probability of transnational healthcare seeking in ethnic minorities who have experienced discrimination.

In this study, all the informants are from developing countries, including Bangladesh, India, Nepal, Sri Lanka and Pakistan, and have travelled back to their home countries for health services. For example, Sadiya was delighted to say everything is so cheap in Bangladesh that she could afford anything. Barsha is mindful of the cost when advising her friends to seek medical treatment in India if their budget is low. In a way, ethnic minorities rely on cross-broader care to escape racial discrimination and inequality that they suffer from the Hong Kong health system.

A South China Morning Post reporter has underscored Hong Kong’s insensitivity to the needs of its minority ethnic communities [19]. My findings concur with Chu’s argument [19], suggesting that local Chinese healthcare providers are culturally insensitive and lack awareness of minority ethnic groups’ cultural traditions, religious beliefs, and taboos. There are 14 Muslims among the 25 informants in the current study, and eight are female. Compared with non-Muslim counterparts, Muslim informants who continue their Islamic beliefs and practices devoutly even though living in Hong Kong, a non-Muslim city, incur more challenges. One such challenge most Muslim women would have encountered is often unable to see a doctor of the same sex. According to the Islamic hierarchy of health-seeking, the Muslim female informants should seek treatment from a Muslim female doctor first, then from a male doctor. If they are unavailable, she could lower the requirement by consulting a non-Muslim female doctor, while seeing a male doctor would be the last option. In Hong Kong’s government clinics or hospitals, patients cannot choose doctors.

Hong Kong is a Chinese-dominant society, with English and Chinese as official languages. The latest census reports that over 96% of its residents speak Chinese, and about 51.9% speak English. Nonetheless, only about 6% of Hong Kong respondents speak English well, and about 1.5% could speak ‘native-like’ English [20], albeit their English lessons begin at kindergarten. The English Proficiency Index 2019 [21] also draws attention to their grave failure in English proficiency. That said, some health providers working in hospitals and general outpatient clinics need to improve their English. Meanwhile, most ethnic minorities do not know Chinese, and some are not even proficient in English. Out of the 25 informants, eight speak only their native tongue. This research study reveals that most informants complained of having difficulty in intercultural health communication, and their English improficiency could subject them to discrimination by healthcare professionals who have no knowledge of ethnic minorities’ native languages. If local Chinese health practitioners misinterpret the semantic meanings in ethnic minorities’ languages, it could lead to an intercultural-health-communication breakdown. Consequently, some ethnic minorities with poor language proficiency prefer assistance from those with the same language and cultural heritage [22]. Chantin stated that most of her friends, speaking very little Chinese, cannot articulate their aliments to Hong Kong doctors, so they prefer returning to their homeland for health care. Like her friends, Farhan, a 30-year-old Pakistani housewife, has been doing just that, for she also likes the idea of *‘you use our own language*’ with her health providers.

In the late spring of 2023, the Hong Kong Secretary of Health, Lo Chung-mau, acknowledged an ongoing shortage of doctors: there were only two doctors for 1,000 people in Hong Kong, whereas Singapore, North America, and European countries had three [23]. Even though the health policy and health system are by regulation to guarantee every citizen equal and affordable access to healthcare services in Hong Kong, the demand for care greatly exceeds the available services. That may challenge the fair allocation of limited resources; for example, triage and queuing add to the existing structural barriers to ethnic minorities’ access to health care. It is not only the local residents begrudging about the long wait and shortage of doctors, as Barsha revealed ethnic minority patients are not satisfied with hospitals for the same reasons and seek treatment overseas. Minhaj, a Bangladeshi male informant, travelled to Bangkok and Singapore for timely medical services. However, Sneha had a different reason for going back to India for treatment because several Hong Kong doctors failed to cure her fever. The negative experiences in the process of healthcare seeking may drive some ethnic minorities to seek health information and healthcare services outside of Hong Kong.

### Cross-border health care: a public health issue

Cross-border care is already a global trend; however, existing health policies in most countries do not admit the existence of health/medical tourism [24]. Practitioners may interpret cross-border health care as a personal choice for seeking alternative therapies or a second medical opinion. Manish did just that, and she sought a second opinion from her family doctor in Pakistan. Policymakers and healthcare providers may view it as a potential opportunity to reduce health and administrative costs, such as employing fewer interpreters. However, not so fast, there are debates regarding the use of cross-border care, i.e., a perceived better option against public health concerns. We should have that in mind when considering the implications of health/medical tourism to the three core principles in health policy analysis, i.e. the cost, quality, and access effects.

When ethnic minorities decide on medical tourism abroad, they must bear all the risks: exposure to new pathogens, liability, and gaps in medical documentation and continuum of care. Research has suggested detailed medical documentation of pre- and post-treatment can facilitate decision-making and treatment pathways, resulting in minimising risk and continuum of care [25]. Regarding the quality of care, though each country has its regulatory policies and professional codes of practice, it is questionable whether patients receive a good standard and appropriate care abroad. There are also ethical and legal concerns regarding how the patients’ country of residence protects its citizens if they encounter malpractice in the process of seeking and utilising medical treatments abroad. Likely, it is the country where the patients reside ending up bearing extra costs and responsibilities for the long-term care of their citizens if anything goes wrong. That is an issue of liability and quality control other than cost containment.

From another perspective, medical tourism violates the principles of solidarity and equity in health care, turning health care into tradable economic services and stratifying patients into affordable and non-affordable groups [24]. In Hong Kong, the triage and queuing strategies adopted in its healthcare finance model [26] may force some patients to seek cross-border health care, despite its objective of equitable access to public healthcare services for every citizen. Thus, this health policy has created healthcare inequality and disparities – rewarding only those who are financially well-off to have a better global network of health care, but not those who are too poor to consider alternative care options other than public healthcare services within Hong Kong.

Despite its well-known and high-standard medical care and facilities, the Hong Kong Government and private sectors are not motivated to promote medical tourism because of the already vast healthcare needs within the local community [27]. In contrast, developing countries in South Asia actively promote medical tourism to generate financial contributions for their governments, consequently gaining a big increase in medical tourists in recent years. For example, India ranks third among wellness-focused countries in the Asia-Pacific and seventh among the 20 wellness tourism markets, generating USD16.3 billion in revenue from medical tourism [28]. Hence, patients from the affordable group seeking medical care from developing countries has been taking advantage of international inequalities for health services at lower costs [29].

Medical tourism violates the principle of global equity in accessing quality care when the task of caring shifts from rich and developed countries to poor and developing countries [30]. That may also exacerbate the healthcare inequalities and health disparities among residents of the countries of destination [31]. When healthcare providers prioritize the medical tourism business over their residents’ health needs, they may redirect resources away from basic healthcare services [32]. Furthermore, medical tourism may cause a brain drain and rural deprivation within destination countries when health professionals move from rural areas to cities, working for higher wages and advanced medical technologies to serve medical tourists [33]. In other words, the health system in developing countries serving medical tourists may be divided into two tiers, i.e., the first tier is for the resourceful residents and tourists who are able to afford the costs of the state-of-the-art medical facilities, while the second tier is for poorer residents without resources. Furthermore, the supply of health professionals and facilities in the destination country is inelastic [32], implying that less-resourced residents may be denied access to limited health resources when competing with medical tourists.

It may, therefore, argue that the ethnic minorities, who have been systemically discriminated against and socially excluded in my study, then become ‘oppressors’ when they compete with the socioeconomically disadvantaged residents for health services of the destination countries. Besides, the reliance on transnational health care may reduce the pressure on departure countries to address problems in their health systems, which subsequently put their citizens at risk [32]. In my study, the key factors forcing my informants to seek cross-border health care are discriminations, language barriers, transnationalism engagement, culturally insensitive practices, and insufficient health professionals from diverse ethnic and cultural backgrounds, as well as factors that are general to all Hong Kong residents, namely seeking cost-effective care and dissatisfaction with local healthcare services. It comes at a price that ethnic minority communities’ use of cross-border health care can shift the caring responsibilities from the government and public healthcare sectors to themselves, relieving the responsible parties of the financial and political pressures to address issues such as a shortage of healthcare workforce and interpreters, etc.; moreover, that feeds the healthcare disparities that inflict upon socioeconomically disadvantaged residents, ethnic minorities, and the local Chinese alike, who have less hope for the crippling Hong Kong healthcare system to improve in the foreseeable future.

### Limitations

The research was undertaken during a period of political instability in Hong Kong due to the protest against the Fugitive Offenders Ordinance. All participants were significantly affected by the social movement, which might have influenced their perceptions and evaluations of Hong Kong’s health care. Besides, the research’s interviews were conducted before the COVID-19 pandemic, which may not have captured the outbreak’s impact on participants’ health-seeking and health-service utilization in Hong Kong and overseas.

### Implications for practice

The present study has found that ineffective health communication between healthcare practitioners and patients, particularly for ethnic minorities, resulted in seeking more cross-border health care. Our findings resonate with one of the ‘soft skills’ of global health competency [34], emphasising the significance of ‘Communication and collaboration’ in healthcare practitioners when interacting with medical tourists. Healthcare professionals should strive to enhance the quality of care and patient satisfaction to promote universal health coverage, ensuring all individuals can access the desired healthcare services without encountering difficulties [35]. In the context of Hong Kong, where the majority of the population is Chinese, it is crucial to improve doctors’ and nurses’ intercultural awareness and sensitivity to foster effective intercultural communication and culturally competent care for ethnic minority groups.

Simultaneously, various stakeholders in Hong Kong have been actively promoting medical tourism, wanting to profit from the region’s high-quality medical scientific research and internationally aligned medical standards. The number of non-eligible persons, meaning those who do not possess a Hong Kong Identity Card, receiving medical services in Hong Kong has increased from 5,971 in 2018–2019 to 6,793 since the COVID-19 pandemic [36]. However, it is projected that Hong Kong will face a shortage of more than 3,000 nurses by 2025 and 5,000 by 2040 [37]. Drawing from the experience of medical tourism in Taiwan [38], the surge of cross-border healthcare users may infringe upon the rights of local residents. Therefore, policymakers should focus on improving hospital infrastructure and increasing the provision of healthcare personnel to mitigate the potential negative impacts on the local population [39].

## Conclusion

Most informants in this study admitted to having sought assistance from their international networks for home remedies, medical advice, and treatments of traditional or Western medicines that are more costly or could not be found/prescribed in Hong Kong. The rationale behind their behaviours is an array of reasons, such as convenience and affordability, still maintaining a close tie to the home country, effective patient-health professional communication, procuring herbal medicine, second medical opinions or treatments that are unavailable in Hong Kong, dissatisfaction with the local health services, etc. To some, in search for a second medical opinion from the medical practitioners, either through a family connection or someone familiar with the informants’ home countries, can be a rational option after they weigh up the health disparity of the host country against the perceived better option in their homeland or other nations. That option can be even more inviting for those with the financial means. On the other hand, humans are social beings — we want to be treated with respect and have our voices heard by the people around us as a sign of acceptance, regardless of race or ethnicity. When ethnic minority informants feel otherwise about their healthcare providers in the host country, they are left with no choice but to go overseas for health care, especially those who always have an affinity for their home country and its people, no matter how long they have been away.

Ethnic minorities’ use of cross-border health care could cause public health concerns, for it relieves the government’s caring responsibilities from healthcare services, so the government might not feel the urgency to fix the failing healthcare system. As for patients, they have to bear the risks of exposure to new pathogens, the extra cost from postoperative complications, gaps in medical documentation and continuity in care, etc. There is an irony in ethnic minorities’ reliance on cross-border health care if they try to evade racial discrimination and inequality in their host country because medical tourism violates the principle of global equity in health care and exacerbates the healthcare inequalities and health disparities in the countries of destination.

### Electronic supplementary material

Below is the link to the electronic supplementary material.

Supplementary Material 1

## Data Availability

The datasets used and/or analyzed during the current study are not publicly available due to the sensitive nature of the data and individual privacy.
