# Supplementary material for: Cross-border healthcare-seeking and utilization behaviours among ethnic minorities: exploring the nexus of the perceived better option and public health concerns
Source: BMC Public Health. 2024 Jun 4;24:1497. doi: 10.1186/s12889-024-18981-1 (PMC11151587; doi:10.1186/s12889-024-18981-1)
Supplement: Supplementary file 1 — Supplementary Material 1 [file 12889_2024_18981_MOESM1_ESM.docx]

**Appendix.1 Consolidated criteria for reporting qualitative studies (COREQ) checklist**

| **No. Item** | **Guide questions/description** | **Reported on Page #** |
| --- | --- | --- |
| **Domain 1: Research team and reﬂexivity** |  |  |
| *Personal Characteristics* |  |  |
| 1. Interviewer/facilitator | Which author/s conducted the interview or focus group? | Page 4 |
| 2. Credentials | What were the researcher’s credentials? E.g. PhD, MD | Page 4 |
| 3. Occupation | What was their occupation at the time of the study? | Page 4 |
| 4. Gender | Was the researcher male or female? | Page 4 |
| 5. Experience and training | What experience or training did the researcher have? | Page 4 |
| *Relationship with participants* |  |  |
| 6. Relationship established | Was a relationship established prior to study commencement? | Page 3 |
| 7. Participant knowledge of the interviewer | What did the participants know about the researcher? e.g. personal goals, reasons for doing the research | Page 3 |
| 8. Interviewer characteristics | What characteristics were reported about the interviewer/facilitator? e.g. Bias, assumptions, reasons and interests in the research topic | Page 4 |
| **Domain 2: study design** |  |  |
| *Theoretical framework* |  |  |
| 9. Methodological orientation and Theory | What methodological orientation was stated to underpin the study? e.g. grounded theory, discourse analysis, ethnography, phenomenology, content analysis | Page 4 |
| *Participant selection* |  |  |
| 10. Sampling | How were participants selected? e.g. purposive, convenience, consecutive, snowball | Page 3 |
| 11. Method of approach | How were participants approached? e.g. face-to-face, telephone, mail, email | Page 3 |
| 12. Sample size | How many participants were in the study? | Page 3 |
| 13. Non-participation | How many people refused to participate or dropped out? Reasons? | No  Page 3 |
| *Setting* |  |  |
| 14. Setting of data collection | Where was the data collected? e.g. home, clinic, workplace | Page 4 |
| 15. Presence of non-participants | Was anyone else present besides the participants and researchers? | Page 4 (Interpreters) |
| 16. Description of sample | What are the important characteristics of the sample? e.g. demographic data, date | Page 3 (Table 1) |
| *Data collection* |  |  |
| 17. Interview guide | Were questions, prompts, guides provided by the authors? Was it pilot tested? | Supplementary materials |
| 18. Repeat interviews | Were repeat interviews carried out? If yes, how many? | No repeat interviews were conducted. |
| 19. Audio/visual recording | Did the research use audio or visual recording to collect the data? | Page 4 |
| 20. Field notes | Were ﬁeld notes made during and/or after the interview or focus group? | Page 4 |
| 21. Duration | What was the duration of the interviews or focus group? | Page 4 |
| 22. Data saturation | Was data saturation discussed? | Page 3 |
| 23. Transcripts returned | Were transcripts returned to participants for comment and/or correction? | No |
| **Domain 3: analysis and ﬁndings** |  |  |
| *Data analysis* |  |  |
| 24. Number of data coders | How many data coders coded the data? | Page 4 |
| 25. Description of the code coding tree | Did authors provide a description of the coding tree? | No |
| 26. Derivation of themes | Were themes identiﬁed in advance or derived from the data? | Page 4 (From data) |
| 27. Software | What software, if applicable, was used to manage the data? | Page 4 |
| 28. Participant checking | Did participants provide feedback on the ﬁndings? | No |
| *Reporting* |  |  |
| 29. Quotations presented | Were participant quotations presented to illustrate the themes/ﬁndings? Was each quotation identiﬁed? e.g. participant number | From page 4 to 6 |
| 30. Data and ﬁndings consistent | Was there consistency between the data presented and the ﬁndings? | Yes |
| 31. Clarity of major themes | Were major themes clearly presented in the ﬁndings? | Yes, from page 4 to 6 |
| 32. Clarity of minor themes | Is there a description of diverse cases or discussion of minor themes? | No |

**Appendix 2. Sampled questions from the interview guide**

1. Tell me who you are- e.g. your ethnic and cultural background, the length of stay in Hong Kong, work, perceived socioeconomic status, and your overall experience in Hong Kong surrounding its social, economic, and political conditions.
2. What were your recent experiences about health seeking in Hong Kong? When and where was it? And who was involved?
3. What is your cultural background and heritage in relation to traditional medicine, complementary and alternative medicine, self-help, self-medication, and transnational healthcare-seeking? By what means?
4. Do you have any international networks beyond Hong Kong to provide you with some health and social care support?
5. Do you have anything you want to highlight health-seeking problems among the ME groups?

Appendix 3. The application of the Four C’s model of thematic analysis

| **Coding** | **Clustering** | **Connecting** | **Constructing** |
| --- | --- | --- | --- |
| Health information definitively we can get from out... I mean, yeah, but maybe we have some family back in Bangladesh. Yeah, we have. So, if I I’m feeling bad or maybe not satisfied with the medicine or I want to recheck that is the good one. It is the appropriate one. I can check all that. I can ask my mom. Could you please ask uncle due to this condition doctor prescribed this medicine is it okay or is there anything better than this or it is the best one here that, that we can check. | Seeking health information from respondents’ connections in their home country | Pre-service: Decision-making and choices in healthcare utilization | Is cross-border health care a personal issue or a public health issue? |
| ‘My sister is in India, my younger sister and she is also a nurse. And, uh, and then sometimes if we feel discomfort, then our, if my child gets sick that I asked how to, what to do like this? |  |  |  |
| I have in my family, I have my brother, he’s a doctor and also, I have some my friend in Bangladesh, they are the doctor in the different, different field. |  |  |  |
| if they know English, it’s easy for me to communicate with them. | Issues related to health-seeking: Language, waiting time, service availability, and cultural differences | Accessibility and barriers |  |
| Most of the friends I heard from them, they tried to, they cannot express well that’s why, that’s why the things will be different. And then most of the friends because of they cannot express well in Hong Kong with the doctor, then they go to their own country to see the doctor. Yea, I heard many. |  |  |  |
| Yeah. It’s a very easy to communicate and get from there. But here maybe you are going to the uh, I mean dispensary, some people don’t understand. Some people will say that what, what, what you need to explain several times this disease and this is so it’s basic like some common what we know that due to cold you need to this have this medicine, fever, this medicine and just common stuff that we collect from Bangladesh. |  |  |  |
| But back in our country, you use our own language, so it’s better you describe everything right to the doctor, and they give you, prescribe you with the medicine. And they think that is better for them to take care, to take that medicine in country you, even though they are here in Hong Kong. And some of them they will… like if they have to do minor operation or something they prefer going back to their country because of the language barrier again. |  |  |  |
| ….if I go to the hospital A&E, it takes us sometimes the two hours, three hours four hours |  |  |  |
| She just keeps on praying to god that today I should get a female doctor |  |  |  |
| All I am saying, they are not satisfied with hospital because long time waiting, not sufficient doctors for all check-ups. So that we can have holiday time we can go Bangkok, Singapore, our countries do the check-up of our health. | Dissatisfaction with healthcare services in the host country | Post-service: Experiences of health-seeking in the host society |  |
| ….a lot of red-tapism…. unable to fulfil all the addition document |  |  |  |
